# Her2+ and b-HCG Producing Undifferentiated Gastric Adenocarcinoma

**DOI:** 10.1155/2014/268919

**Published:** 2014-10-01

**Authors:** Sahar Eivaz-Mohammadi, Fernando Gonzalez-Ibarra, Waheed Abdul, Omer Tarar, Khurram Malik, Amer K. Syed

**Affiliations:** ^1^Jersey City Medical Center, Department of Internal Medicine, 355 Grand Street, NJ 07302, USA; ^2^St. George's University School of Medicine, Jersey City Medical Center, Department of Internal Medicine, 355 Grand Street, Jersey City, NJ 07302, USA; ^3^Jersey City Medical Center, 355 Grand Street, Jersey City, NJ 07302, USA

## Abstract

A 25-year-old Hispanic female with a history of anemia, schizoaffective disorder, and psychosis was admitted for anemia associated with fatigue, weakness, shortness of breath, night sweats, weight loss, and abdominal and lower back pain for the past two months. On routine management, she was found to have a positive serum b-HCG of 80.4 (0–5 mIU/mL) but the patient denied any sexual activity in her life. During her admission, U/S of the pelvis was noncontributory. CT angiogram of the chest was significant for prominent mediastinal and hilar lymph nodes, diffusely thickened stomach suggesting gastric malignancy with multiple hypoenhancing lesions in the liver and diffuse lytic lesions in the spine and sacrum suspicious for metastatic disease. The MRI of the abdomen confirmed the CT angiogram findings. After these findings, EGD was performed which showed lesions in the antrum, body of the stomach, fundus, and cardia on the lesser curvature of the stomach body correlating with carcinoma. The biopsy was positive for Her2, b-HCG producing poorly differentiated gastric adenocarcinoma. Patient underwent one successful round of chemotherapy with Taxotene, Cisplatin, and 5-FU for Stage IV gastric adenocarcinoma.

## 1. Introduction

Early gastric cancer is defined as an adenocarcinoma which is limited to the gastric mucosa and the submucosa, regardless of regional lymph nodes involvement [1]. According to Lauren's classification, gastric cancer is classified morphologically into three categories: undifferentiated (diffuse), well differentiated (intestinal) (Lauren), and mixed subtype. The classification is based on the cancer's morphology, epidemiology, pathogenesis, and genetics [2].

Gastric adenocarcinoma has a higher incidence rate in Asian, Hispanic, and African American as compared to the Caucasian population [3]. Recent studies have shown that there has been an increased incidence in young adults in the United States. It has the second leading global cancer mortality with incidence rate of more than one million cases per year [3].

Undifferentiated gastric carcinoma is characterized as poor histological presentation and presence of signet-ring cell carcinoma [2]. It is very rare to have an increased b-HCG and Her2+ expression associated with undifferentiated adenocarcinoma. We report a case featuring young Hispanic female with elevated B-HCG and Her2+ undifferentiated gastric adenocarcinoma.

## 2. Case Report

A 25-year-old Hispanic female with a history of schizoaffective disorder, psychosis, and iron deficiency anemia was sent to the ED by her PMD when she was found to be severely anemic on routine blood testing associated with fatigue, weakness, shortness of breath, night sweats, 7-8 lb unintentional weight loss, and abdominal and lower back pain for the past two months. In addition to these symptoms, she was having minor postoperative complications of dysphagia and nausea with vomiting after meals since her tracheal cyst removal one month prior. She denied any hemoptysis, hematuria, melena, menorrhagia, overuse of NSAIDs, any toxic habits, or recent sexual activity. Her last menstrual period was two weeks before this admission. Her family history is significant of grandmother diagnosed with breast cancer at the age of 60.

At the time of this admission, the patient was alert, awake, oriented, afebrile, and hemodynamically stable except for tachycardia. She appeared weak and tired. Despite complaints of weakness, physical examination was only pertinent positive to conjunctival pallor. Stool guaiac test in the ER was negative.

In the ED, the patient's complete blood count and serum chemistry were within normal limits except for Hemoglobin of 6.7 and Hematocrit of 19.9 compared to her approximate baseline of 11 and 32, respectively. Patient was transfused with 1PRBC while in the ED. Although the urine b-HCG was negative, her serum b-HCG was 80.4 (0–5 mIU/mL). On further questioning, patient mentioned that she has never had any sexual intercourse. Initially, it was thought that she was pregnant and an ultrasound of the pelvis was performed; the results were a normal anterverted uterus with endometrial thickness of 3 mm and no lesions or mass was seen. Subsequently, patient was admitted for further evaluation.

**Figure 1:**
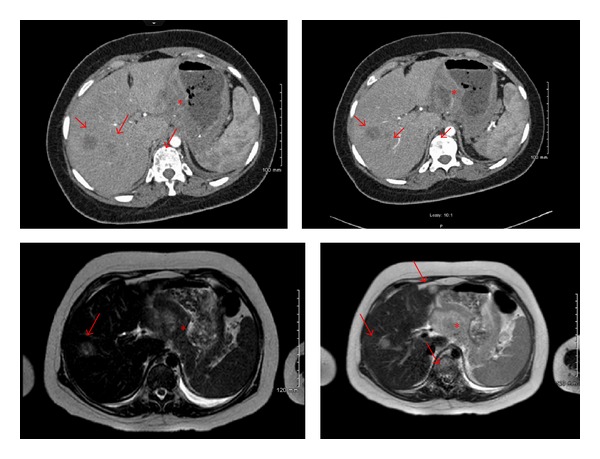
Incidental finding of thickening of stomach lining (asterisk), multiple liver and spinal metastases (arrows) found in the CT with contrast angiogram of the chest.

**Figure 2:**
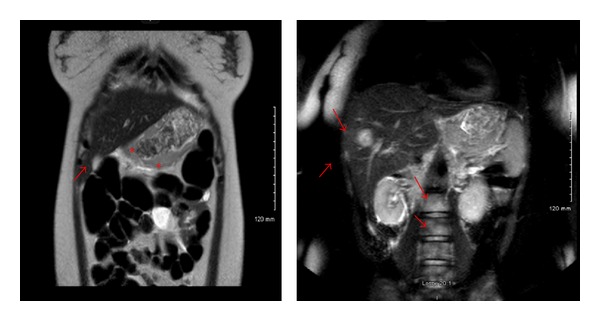
MRI with and without contrast of the abdomen showing at least two liver metastases, stomach thickening, and spinal metastasis.

During her admission, CT angiogram with IV contrast of the chest was ordered to rule out pulmonary embolism which was negative for pulmonary embolism but significant for prominent mediastinal/hilar lymph nodes, diffusely thickened stomach suggesting gastric malignancy with multiple hypoenhancing lesions in the liver, diffuse lytic lesions in the spine, and sacrum suggesting metastatic disease (Figure 1). MRI of the abdomen and pelvis was ordered by Ob/Gyn for further. The MRI of the pelvis was negative but the MRI of the abdomen confirmed the CT findings of marked gastric thickening with mass-like 3 cm × 5 cm structure in the lesser sac, multiple liver masses, retroperitoneal lymphadenopathy, and bone lesions (Figure 2).

**Figure 3:**
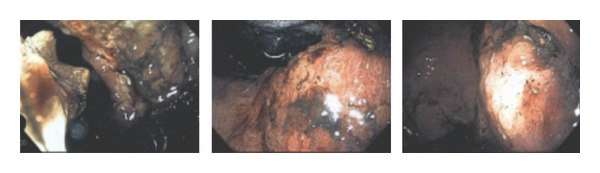
Esophagogastroduodenoscopy showing lesions in the antrum, body of the stomach, fundus, cardia, and the lesser curvature of the stomach body consistent with carcinoma.

**Figure 4:**
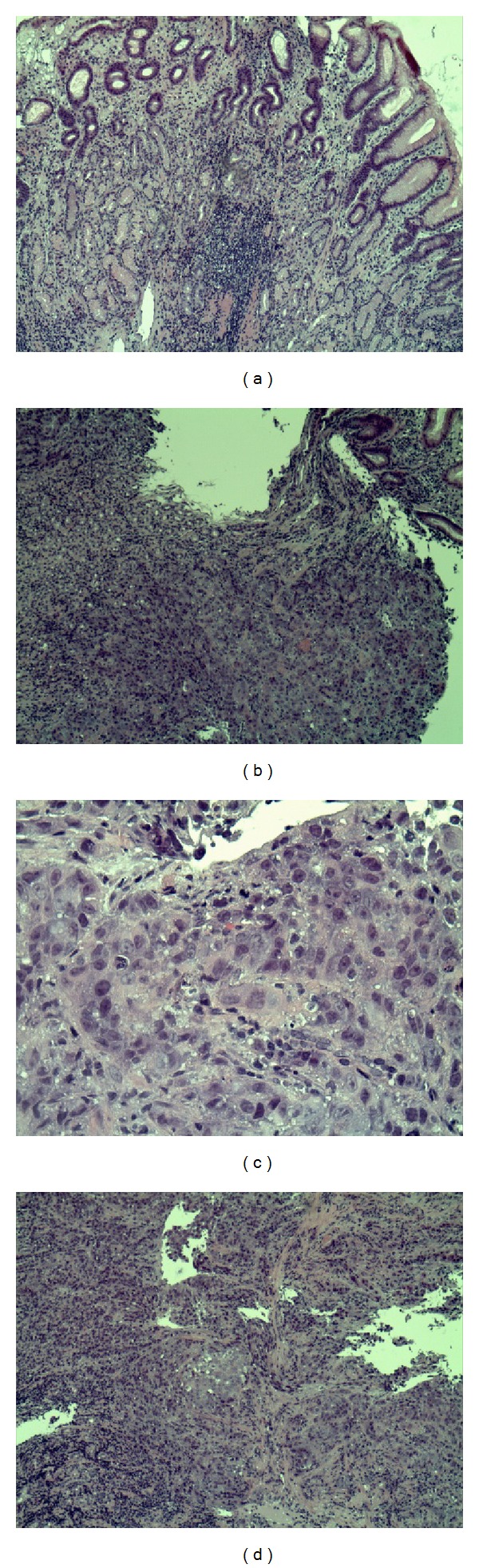
Histopathology of gastric cells. (a) Shows normal gastric cells consisting of mucous cells, parietal cells, and chief cells. (b), (c), and (d) Show undifferentiated adenocarcinoma gastric tumor cells.

**Figure 5:**
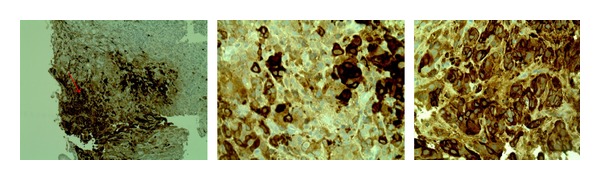
Histopathology of undifferentiated gastric tumor cells stained positive for b-HCG.

**Figure 6:**
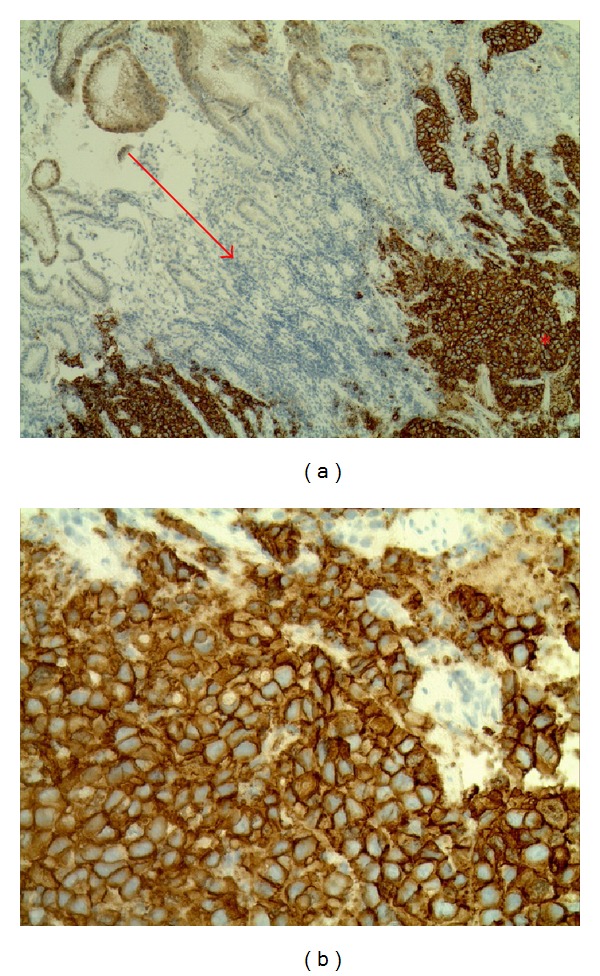
(a) The arrow indicates normal gastric cells (unstained) and asterisk shows Her2+ stained cancerous cells. (b) Shows Her2+ stained gastric cancerous cells.

After discussing the findings with the patient, she agreed to undergo esophagogastroduodenoscopy to examine the underlining of the stomach and obtain a biopsy. EGD report showed lesions in the antrum, body of the stomach, fundus, and cardia on the lesser curvature of the stomach body correlating with carcinoma (Figure 3). The biopsy was positive for b-HCG and Her2 producing poorly differentiated gastric adenocarcinoma (Figures 4, 5, and 6). The tumor markers CEA, CA 19-9, and CA 125 were all negative. CA 72-4 marker was not tested.

Patient was reevaluated after the EGD and successful 1 round of chemotherapy (Taxotene, Cisplatin, and 5-FU) for Stage IV gastric adenocarcinoma was started. At that time, the histopathology of the Her2 staining was pending; therefore, herceptin was not started.

After the first round of chemotherapy, patient was transferred to Mount Sinai medical center as per her and her family members' wishes.

Currently, patient is undergoing chemotherapy with taxotene, 5-FU, Oxaloplatin and leucovorin, and herceptin. She is scheduled to undergo genetic testing as her grandmother had breast cancer in her 60s.

## 3. Discussion

Gastric cancer is twice more common in males than in females with the highest incidence in South America, Eastern Asia, and Eastern Europe and lowest in North America [3]. Multiple published articles have shown that some of the established risk factors are family history,* H. pylori* infection, tobacco usage, fatty diet, and pernicious anemia; however, its etiology is relatively unknown [4]. Sign and symptoms of gastric cancer are nonspecific but usually associated with weight loss, fatigue, dyspepsia, early satiety, and melena [4]. In majority of the cases, physical examination is relatively benign [2].

Family history of cancers such as familial adenomatous polyposis (FAP), Li-Fraumeni syndrome, prostate cancer, hereditary nonpolyposis colorectal carcinoma (HNPCC), and Peutz-Jeghers syndrome has a strong association with gastric cancer [5]. Also, multiple other studies have shown that BRCA2 mutation but not BRCA1 is correlated with gastric cancer [6, 7].

Approximately, 95% of gastric cancers are adenocarcinomas where 53% and 33% of them are classified as differentiated and diffuse type, respectively [8]. The intestinal-type carcinoma precedes with multifocal atrophic gastritis that is more common in elderly men [9]. Undifferentiated (diffuse) gastric adenocarcinoma usually occurs predominantly in female patients younger than 50 years old and has a poorer prognosis [8]. Intestinal-type adenocarcinoma tumor cells were described as irregularly well-demarcated tubular structures, harboring pluristratification, multiple lumens, and reduced stroma [10].

Diffuse type tumors exhibit deep infiltration of the stomach wall and the histologic precursor lesion is usually not identifiable [10]. Intestinal-type tumors show gland formation cells that are discohesive and secrete mucus delivering in the interstitium, producing large pools of mucus/colloid [10]. It is found to be commonly more extensive, invasive, and associated with wide serosal invasion and frequent metastasis predominantly to the liver and peritoneum than the intestinal-type tumor [4]. In many cases, hematogenous spread of the tumor leading to metastasis to the bone, lungs, and brain has been reported [9]. The prognosis of this type of tumor is poor, even if radical gastrectomy and extended lymph node dissection are performed [9].

One of the main differences between the two types of gastric tumor was the onset of peritoneal recurrence, which was observed in 34% in diffuse type cases compared to 9% of intestinal-type cases [9]. The recurrences of intestinal-type tumors were mainly locoregional or hematogenous [9]. The main morphological difference between intestinal and diffuse type gastric carcinoma is a presence of defective intercellular adhesion protein (E-cadherin, CDH1) in diffuse type [11]. This allows the cancerous cells to grow and invade the neighboring structures faster. Also, genetic studies have identified two specific genomic subtypes, G-INT (intestinal) and G-DIF (diffuse) corresponding to intestinal and diffuse type, respectively [12]. Tan et al. have shown that both of these intrinsic subtypes arise from unsupervised cell line and both have distinct patterns of gene expression [12]. Kaplan-Meier curves have shown that G-INT has a better prognosis than G-DIF. G-DIF is susceptible to Cisplatin and G-INT tumor cells are more susceptible to 5-FU and Oxaloplatin [12].

Commonly, b-HCG is not found as a paraneoplastic hormone in different cancers especially gastric cancers. There are three types of b-HCG: free, pituitary, and hyperglycosylated b-HCG. Advanced cancers such as teratomas, hydatidiform, choriocarcinomas, germ cell tumors, leukemia, and lymphomas release free b-HCG [13]. Several studies have indicated that this type of b-HCG promotes cell invasion and metastasis. Hyperglycosylated b-HCG is produced by the synctiotrophoblast and interacts with the blastocyst to promote the maintenance of corpus luteum and growth of placenta. Pituitary b-HCG is associated with the female's menstrual cycle [13]. Undifferentiated gastric adenocarcinoma associated with b-HCG is very rare and only a handful of cases are reported in the literature.

Normally gastric adenocarcinoma is not associated with high levels of b-HCG but in female reproductive patients presenting with vague signs and symptoms of gastrointestinal issues, gastric malignancy should be ruled out after pregnancy test showed negative results.
